# Deletion at 12q12 increases the risk of developmental delay and intellectual disability

**DOI:** 10.1111/ahg.12279

**Published:** 2018-08-29

**Authors:** Ying Weng, Xiaoping Luo, Ling Hou

**Affiliations:** ^1^ Department of Paediatrics Tongji Hospital, Tongji Medical School, Huazhong University of Science and Technology Wuhan Hubei China

**Keywords:** 12q12 deletion, copy number variant, growth retardation

## Abstract

Single‐nucleotide polymorphism (SNP) arrays have been widely used to identify novel genomic imbalances. Many of these genomic imbalances have been confirmed to interact with developmental delays, intellectual disabilities (IDs), and congenital defects. Here, we identify a Chinese girl with a 3.18‐Mb deletion at 12q12 (human genome build 19: 43,418,911‐46,601,627) who showed postnatal growth delay, low‐set ears, small hands and feet, widely spaced nipples, and blue sclerae. Deletions at 12q12 are extremely rare chromosomal imbalances; only four cases involving a deletion of this type have previously been reported. In these five sporadic cases, all of the patients exhibited developmental issues accompanied by different degrees of ID. A review of DECIPHER patient data revealed an additional six cases involving genomic deletion at 12q12. Many of the patients in these cases exhibited developmental delay and ID. When these patients were included, 91% and 73% of individuals with a deletion in this chromosomal region presented with developmental retardation and ID, respectively. Database searches indicated that this copy number variant (CNV) has not been found in normal humans. Therefore, we suggest that a CNV in this region is a risk factor for developmental retardation and ID.

## 1 INTRODUCTION

Growth deficiency occurs in association with many different chromosome abnormalities, including microdeletions and microduplications (Batey et al., 2014; Capalbo, Rienzi, & Ubaldi, 2017; Li et al., 2017). Chromosomal microarray analyses (CMAs) have revealed a large number of copy number variants (CNVs), which refer to a length of DNA larger than 1 kb with a different copy number than that observed in normal humans, on chromosomes. A multitude of CNVs have been found in patients with clinical phenotypes such as autism, developmental delay, and intellectual disability (ID), and many of these CNVs have been confirmed to be associated with diseases (Lowther, Costain, Baribeau, & Bassett, 2017; Takumi, & Tamada, 2018). CNVs that have not been reported in normal humans in database records and do not overlap with genomic regions known to be associated with disorders are temporarily classified as CNVs of unknown significance. Building connections between these novel CNVs and clinical phenotypes remains a considerable challenge. Such investigations are essentially dependent on case accumulation, which requires long‐term efforts.

Recently, we encountered a 3‐month‐old girl with a 3.18‐Mb deletion at chromosome 12q12, a CNV of unknown clinical significance. A literature review revealed that 12q12 deletions are extremely rare; only four cases involving such deletions have previously been reported (Carlsen, Frengen, Fannemel, & Misceo, 2015; Failla et al., 2008; Miyake et al., 2004; Tonoki, Saitoh, & Kobayashi, 1998). Although most of these cases were sporadic, the patients in these cases shared certain clinical characteristics. The girl we encountered exhibited growth retardation and ID with the deletion at 12q12. Although 12q12 deletions have previously been observed, the present study is a new demonstration in which an association between this type of deletion and developmental delay has been established. Based on the different rates of occurrence of 12q12 deletion in patients and normal individuals, we suggest that 12q12 deletion is a novel risk factor for developmental delay.

## 2 CLINICAL REPORT

The Chinese girl we encountered was born to a 34‐year‐old mother and a 34‐year‐old father after 40 weeks of gestation by spontaneous vaginal delivery. She was the second child in the family, and her 12‐year‐old sister was normal. Both parents were healthy, and consanguinity was denied. The mother had gestational diabetes mellitus and hyperthyroidism. No intrauterine growth retardation was reported. The patient's birth weight was 3.14 kg (50th–75th centile), and her birth length was 46 cm (< 3rd centile). Her Apgar score was unknown. There was second‐level meconium staining of the amniotic fluid at her birth. She stayed in the neonatal intensive care unit for 8 days because of aspiration of amniotic fluid. Physical and laboratory examinations revealed congenital heart disease, an atrial septal defect, one epulis on the lower half of the oral cavity, hypocalcemia (serum calcium level, 1.52 mmol/L), and high levels of aspartate aminotransferase and uric acid. In addition, the patient was sensitive to egg white and had feeding difficulties.

**Figure 1:**
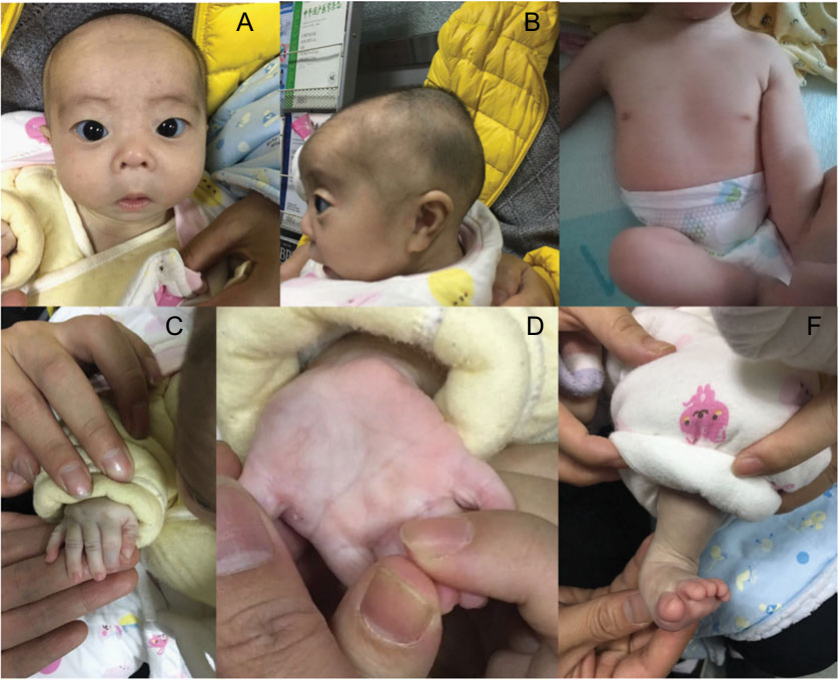
Appearance of the patient at the age of 3 months. A, Facial features, including upslanting palpebral fissures, a broad nasal bridge with anteverted nares, and blue sclerae. B, Lateral view showing a short neck and low‐set, large ears. C, Small hands and fifth finger clinodactyly. D, Intersecting palms. E, Widely spaced nipples. F, Small feet [Color figure can be viewed at http://wileyonlinelibrary.com]

Other observed anomalies included mildly dysmorphic features (Figure 1 A–F), such as a short neck; upslanting palpebral fissures; large, low‐set ears; a broad nasal bridge with anteverted nares; downturned corners of the mouth; widely spaced nipples; fifth finger clinodactyly; small hands (total hand length, 6.5 cm); small feet (total foot length, 7 cm); and blue sclerae.

After discharge, the patient had growth issues. Her weight at 1 month was 3.2 kg (< 3^rd^ centile), her length was 46 cm (< 3^rd^ centile), and her head circumference was 35.5 cm (25th–50th centile). When she was 3 months old, her weight was 3.2 kg (< 3^rd^ centile), her length was 49 cm (< 3^rd^ centile), and her head circumference was 36 cm (< 3^rd^ centile). At 6 months, her weight was 3.3 kg (< 3rd centile), her length was 52 cm (< 3rd centile), and her head circumference was 36 cm (< 3rd centile). At 8 months, her weight was 3.6 kg (< 3rd centile), her length was 53 cm (< 3rd centile), and her head circumference was 38 cm (< 3rd centile). During these 8 months, her weight had only minimally increased, and increases in her height and head circumference markedly lagged behind those of normal infants.

## 3 GENETIC TESTING

This study was approved by ethics committees of our hospital, and consent was obtained from the patient's parents. G‐banding with 300 to 400 bands performed on leukocytes from the patient's peripheral blood revealed a balanced translocation. The patient's karyotype was 46,XX, t (12;14) (q12;q24), which is t (12; 14) (12pter→12q12::14q24→14qter; 14pter→14q24::12q12→12qter). No imbalance at the breakpoint of the translocation was detected by karyotype analysis.

CMA was performed on a CytoScan 750K Array (Affymetrix, CA) in accordance with the manufacturer's instructions. Genomic DNA was extracted from peripheral blood and isolated via standard procedures using a QIAamp DNA Blood Mini Kit (Qiagen, Hilden). PCR was performed on a 9700 thermal cycler (AB, Singapore). CMA revealed a 3.18‐Mb interstitial deletion at 12q12 (43,418,911‐46,601,627). No imbalance at the breakpoint of 14q24 was detected. The reciprocal translocation and the deletion at 12q12 were *de novo*.

## 4 DISCUSSION

**Figure 2:**
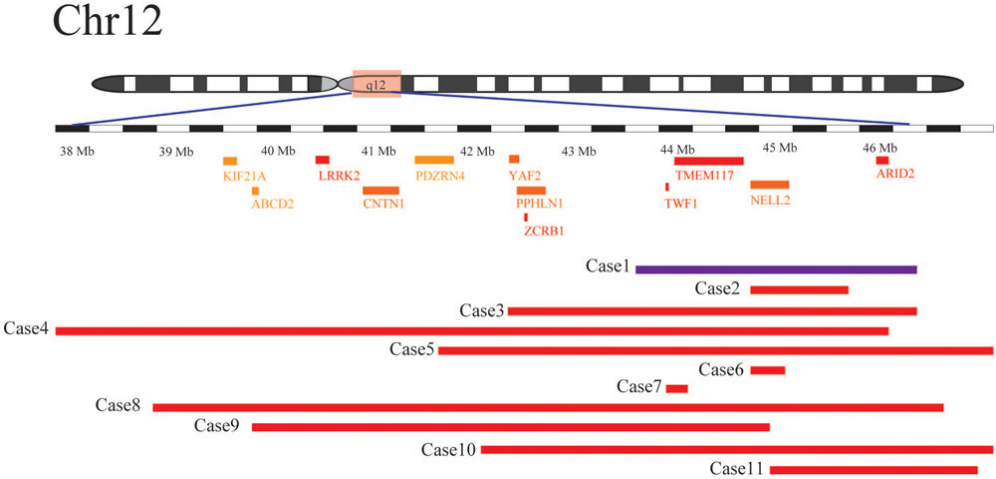
Genes with high HI scores in the deleted 12q12 regions (modified from the DECIPHER genome browser: https://decipher.sanger.ac.uk) in our patient and 10 other patients. Case 1 is the girl we have reported, case 2 was reported by Carlsen et al. in 2015, case 3 was reported by Failla et al. in 2008, and cases 4 and 5 involved patients 1 and 2 (first reported by Tonoki et al., 1998), respectively, of the patients reviewed by Miyake et al. in 2004. Cases 5 to 11 were obtained from the DECIPHER database [Color figure can be viewed at http://wileyonlinelibrary.com]

We observed a patient with a 3.18‐Mb deletion at 12q12 who exhibited severe growth issues. The 3.18‐Mb loss of 12q12 was possibly associated with one of the breakpoints of the translocation. This microdeletion represents a novel chromosomal imbalance that was not reported in genetic data from more than 7000 normal humans recorded in the Database of Genomic Variants. The deletion gene content in our patient (case 1) is presented in Figure 2. This portion of the chromosome includes 11 genes: *ADAMT* (haploinsufficiency score, HI score: 68.03%), *PUS7L* (68.50%), *IRAK4* (45.47%), *TWF1* (19.19%), *TMEM117* (12.80%), *NELL2* (20.20%), *DBX2* (58.99%), *ANO6* (55.40%), *ARID2* (11.01%), *SCAF11* (49.38%), and *SLC38A1* (41.45%). Because the cut‐off value of HI score is 25%, four of these genes, *TWF1*, *TMEM117*, *NELL2*, and *ARID2*, with high haploinsufficiency (HI) scores indicate that a heterozygous deletion is relatively likely to induce loss of function.

Loss‐of‐function mutations of *ARID2* cause an ID syndrome that was reported in 2015 (Shang et al., 2015). SWI/SNF chromatin modifier has been related to neurodevelopmental disorders, including ID and autism. ARID protein is the SWI/SNF subcomplex. Shang et al. reported four patients with mutations of *ARID2*, who all showed ID and developmental delay, and one of the patients sharing the same phenotype with our patient had atrial septal defect. Deletion of *ARID2* possibly caused the congenital heart defect of our patient.

NELL2 protein is neural epidermal growth factor‐like‐like 2. Previous study has been proved that NELL2 protein plays a part in neuronal proliferation, differentiation, synaptic formation, and plasticity during development and postnatal life (Choi et al., 2014). Jeong et al. investigated NELL2‐regulated feeding behavior. A block of NELL2 production led to a decrease in daily food intake followed by a loss in body weight (Jeong et al., 2017). According to the parents’ report, our patient showed feeding difficulties in the first 3 months. The girl's appetite was less than 50 mL of milk per day. This might be one of the reasons for the growth deficiency.

Twinfilin 1 encoded by *TWF1* is an actin monomer–binding protein. Twinfilin 1 was reported to be involved in ocular coloboma (Rainger et al., 2017). *TMEM117* knocked down would alter homeostasis toward cell death and *TMEM117* RNAi‐facilitated apoptotic cell death (Tamaki et al., 2017). So far no paper published has reported that *TWF1* or *TMEM117* is connected with developmental delay.

Ten cases involving deletion at 12q12 have previously been reported in the literature (Table 1) and the DECIPHER database (Table 2). Although phenotypes of developmental delay and ID were often observed, associations between 12q12 deletion and such phenotypes have not previously been established, and this microdeletion has not been regarded as pathogenic.

**Table 1:** Summary of clinical features of five previously reported patients compared with those of the new patient described in this report

	Case 1	Case 2	Case 3	Case 4	Case 5
Patient information	Current case	Carlsen et al., 2015	Failla et al., 2008	Miyake et al., 2004 (1)	Miyake et al., 2004 (2), Tonoki et al., 1998
Sex	Female	Male	Male	Male	Male
Deleted cytoband	12q12	12q12	12q12	12q11‐q13	12q12‐q13.2
Age at evaluation	3 mo	10 yr	10 yr	20 mo	2 yr
IUGR	−	−	−	−	+
Growth retardation	+	+	+	+	+
ID	Moderate	Moderate	Moderate	Moderate	Severe
OFC (< 3^rd^ centile)	+	−	+	NA	+
Small hands and feet	+	+	+	+	+
Large and low‐set ears	+	+	+	+	+
Eye abnormalities	Blue sclerae	Strabismus	Strabismus, myopia	Strabismus, myopia	Strabismus, blepharoptosis
Palpebral fissures	Up	Down	Horizontal	Up	Down
Nose (broad nasal bridge or anteverted nostrils)	+/+	+/+	+/−	+/+	+/+
Mouth	Small, downturned corners, long philtrum	Wide mouth, long flat philtrum	Downturned corners, long flat philtrum	Small, downturned corners	Small, downturned corners; long philtrum; cleft palate
Fifth finger clinodactyly	+	+	−	NA	+
Widely spaced nipples	+	+	+	+	+
Cardiologic anomalies	+	+	+	+	−

Legend: ID: intellectual disability; IGR: intrauterine growth retardation; NA: not assessed

**Table 2:** Clinical features of ten reported cases involving genomic loss at 12q12 in the DECIPHER database

	Case 6	Case 7	Case 8	Case 9	Case 10	Case 11
**ID**	139	250361	257543	259419	285576	349958
**Size of 12q12/ inheritance**	497.5 kb/ unknown	166.44 kb/ inherited	8.08 Mb/ unknown	5.34 Mb/ de novo	6.49 Mb/ de novo	2.22 Mb/ de novo
**Coordinates (hg19)**	44866421‐ 45363917	44126853‐ 44293297	38805678‐ 46882370	39933990‐ 45269105	42264141‐ 48750492	45161827‐ 47383364
**Additional CNVs**	Loss chr12: 16851561‐ 22736846	None	None	None	None	Gain chr15: 22770421‐ 23288350
**Developmental features**	Speech and language development delay	No information	Proportionate short stature	Delayed speech and language development, motor delay	Delayed speech and language development	Mild global development delay, short stature
**Intellectual disability**	+	+	+	No information	No information	No information
**Facial /Cranial dysmorphisms**	Prominent nasal bridge	Facial abnormality	No information	No information	No information	Mild microcephaly
**Other clinical features**	No information	Abnormal hair pattern	Autism, precocious male puberty	No information	Cleft palate	No information

Carlson et al. reported a boy with a 1.13‐Mb deletion at 12q12 who exhibited growth delay, psychomotor delay, concentration and attention deficits, and an intelligence test score typical of children who are 2 to 3 years younger (case 2, Table 1). In the deleted region, only three protein‐coding genes were detected: *ANO6*, *DBX2*, and *NELL2*. Therefore, the authors concluded that these three genes are involved in developmental delay.

Six additional cases (two cases with no provided clinical information were excluded) of 12q12 deletions were found in the DECIPHER database (cases 6–11, Table 2). Three individuals had *de novo* 12q12 deletions, one individuals had inherited deletions, and inheritance information was not available for the remaining cases. The fact that the parent of one individual (Adam, Mehta, Knight, Hall, & Rossi, 2010) was also affected by a 12q12 deletion suggested that this 12q12 deletion had likely cosegregated with the reported phenotypes in that family.

Developmental delay to varying extents and ID or mental retardation were observed in 91% (10 of 11) and 73% (8 of 11), respectively, of all 11 reported cases involving 12q12 deletion. Three patients were diagnosed with autism spectrum disorder, and two patients had concentration and attention deficits. Craniofacial dysmorphism was noted in our patient and in 5 of the individuals described in the literature and the DECIPHER database. Thus, at least 55% (6 of 11) of individuals with a reported 12q12 deletion exhibited various craniofacial dysmorphisms.

Certain reported individuals carried additional genomic variations that could have affected their overall phenotypes. Individual 6 had a 5.89‐Mb deletion at 12p12.3 with possible clinical significance. There was additional genomic imbalances in case 11 that was likely benign or of uncertain clinical significance.

In the 11 individuals described here, the deleted segments were partially or completely in 12q12. All dosage‐sensitive genes in this region are listed in Figure 2.

In two cases (cases 6 and 7), the deleted segments were smaller than 1 Mb and included at least one dosage‐sensitive gene; in particular, the affected genes included *NELL2* in individual 6 and *TWF1* and *TMEM117* in individual 7. These findings suggest that *NELL2* and either *TWF1* or *TMEM117* play roles in development. *NELL2* mRNA levels were also found to be decreased in patient 2 (Carlsen et al., 2015). Certain genes in 12q12 have the same development‐related functions. It appears likely that 12q12 acts as a functional unit in development.

In summary, we presented 11 individuals with 12q12 deletion, including one new subject. Our analysis suggested that a 12q12 microdeletion increases risk for developmental delay and ID. In conclusion, 12q12 deletion should be added to the database of pathophysiological genomic alterations that induce developmental delay, which will be helpful for counselling and management of the patients with developmental delay.

## CONFLICT OF INTEREST

The authors declare no conflicts of interest.
